# Prevalence of high-risk HPV types and associated genital diseases in women born in 1988/89 or 1983/84 – results of WOLVES, a population-based epidemiological study in Wolfsburg, Germany

**DOI:** 10.1186/1471-2334-13-135

**Published:** 2013-03-13

**Authors:** Karl Ulrich Petry, Alexander Luyten, Annika Justus, Angelika Iftner, Sarah Strehlke, Axel Reinecke-Lüthge, Elisabeth Grunwald, Renate Schulze-Rath, Thomas Iftner

**Affiliations:** 1 Klinikum Wolfsburg, Frauenklinik, Schwerpunkt gynäkologische Onkologie, Sauerbruchstr.7, Wolfsburg, 38440, Germany; 2 Conreso GmbH, Klinische Forschung, Neuhauser Straße 47, Munich, 80331, Germany; 3 Universitätsklinikum Tübingen, Institut für Virologie, Sektion Experimentelle Virologie, Elfriede Aulhorn Str. 6, Tübingen, 72076, Germany; 4 Klinikum Wolfsburg, Institut für Pathologie, Sauerbruchstr. 7, Wolfsburg, 38440, Germany; 5 Praxis für Frauenheilkunde, Kaufhofpassage 5-7, Wolfsburg, 38440, Germany; 6 Sanofi Pasteur MSD GmbH, Paul Ehrlich Strasse, Leimen, 69181, Germany

**Keywords:** High-risk HPV, HPV16, CIN, HPV vaccination

## Abstract

**Background:**

High-risk human papilloma virus (HR-HPV) infection is associated with the development of cervical cancer. HPV vaccination reduces the risk of developing malignant lesions and is expected to change the dynamics of HPV transmission. Data from non-vaccinated women may provide an important benchmark to allow the impact of HPV vaccination programs to be assessed.

This study was designed to prospectively determine the changing dynamics of HR-HPV infection and associated genital diseases in young women, most of whom were non-vaccinated.

**Methods:**

Data from a population-based cohort study, comprising women of two predefined birth cohorts (women born in 1983/84 or 1988/89), were analyzed between 19 October 2009 and 31 December 2010 to determine risk factors for high-risk HPV infection and the association between specific HR-HPV types and atypical Pap smear test results. HPV status was determined by Hybrid Capture 2 (HC2) assay and genotyping.

**Results:**

The prevalence of HR-HPV was 22.8% in the 1983/84 cohort (150/659) and 23.7% in the 1988/99 cohort (142/599). Only the number of sexual partners was a significant risk factor for HPV infection (odds ratios 22.687 and 6.124 for more than five versus one partner 84 cohort,/84 and 1988/89 cohorts, respectively) in multivariate analysis. HPV16 positive-women were significantly more likely to have abnormal Pap smears of any degree than HPV16-negative women (22.0% versus 3.61%, p < 0.0001 for the 1983/84 cohort and 9.09% versus 2.52%, p = 0.0482 for the 1988/89 cohort). CIN3 was diagnosed in six women 84 cohort,/84 cohort and two in the 1988/89 cohort. All women with CIN3 tested positive for HC2-HR and all six CIN3 cases 84 cohort,/84 cohort tested positive for HPV16. In the 1988/89 cohort, the rate of HPV16 infection was significantly lower in vaccinated than non-vaccinated women (1.59% versus 8.88%; p = 0.003).

**Conclusions:**

HR-HPV infection was highly prevalent in both cohorts and associated with an increased risk of abnormal Pap smears and biopsy proven CIN2+. HPV16 infection was associated with a high risk of clinically relevant lesions. HPV vaccination significantly decreased the risk of HPV16 infection.

## Background

Thirteen types of human papilloma virus (HPV) are associated with the development of cervical cancer and are designated as high-risk (HR) [1]. Among the HR types, HPV types 16 and 18 are associated with 70% of all cervical cancer cases [2]. Prophylactic vaccines directed against the HR types 16 and 18 prevent infection of the cervical epithelium and other squamous epithelia, decreasing the development of premalignant lesions [3,4]. The best results of prophylactic vaccination are achieved by vaccinating women before they become sexually active and genitally infected [5].

Vaccination programs are expected to change the dynamics of HPV transmission [6] and, therefore, it is imperative to monitor the burden of HPV infections and associated diseases in young women who had reached the age of 18 years or more prior to the implementation of routine vaccination. German groups have already published epidemiological data from HPV and cervical cancer screening trials [7-10], but most of these trials excluded women younger than 30 years of age. An observational study reported by Iftner and colleagues included 1692 females aged 10–30 years, but the one-time cross-sectional analysis provided only limited clinical information [10]. In Germany, a national, reimbursed annual screening program with Pap (Papanicolaou) smear testing starts for women at age 20 years and data show that atypical screening results are reasonably common in women aged 20–25 years and even more so in those aged 25–30 years [11]. In the German screening program, women have to attend a gynecologist in private practice. Since 2006, gynecologists in private practice in Wolfsburg have formed an experienced screening and referral network with the Klinikum Wolfsburg to improve cervical cancer prevention. This collaboration has provided a final opportunity to prospectively study the dynamics of HPV infection and associated genital diseases in a population comprising mostly non-vaccinated young women in Wolfsburg, Germany. This paper reports the results on the prevalence of HR-HPV types and associated diseases. Data for low-risk (LR)-HPV are published separately [12].

## Methods

The Wolfsburg HPV Epidemiological Study (WOLVES) is a prospective, population-based surveillance study on the prevalence and incidence of HPV infections and associated diseases in women of three predefined birth cohorts. All women born in 1983/84, 1988/89, and 1993/94 with a first residency in Wolfsburg, Germany (population 123,000), will be invited to participate. This analysis includes baseline data for the 1983/84 and 1988/89 cohorts.

### Study population

The residents’ registration office provided a list of women with a first residency in Wolfsburg born in either 1983/84 or 1988/89 and all 2850 women were invited by letter to attend cervical cancer screening. The invitation included information on the study objectives and made it clear that participation was voluntary. To be recruited, women had to attend one of 20 gynecologists in private practices in the city of Wolfsburg for routine Pap smear screening, according to the standard screening concept in Germany. All participants gave written consent and the study was approved by the ethics committee of the physicians’ association of Lower Saxony in Hannover, Germany.

Participants completed a short, standardized questionnaire in the private practice. The questionnaire included questions on education, birth country, marital status, pregnancies, parity, contraception, smoking, number of sexual partners, age at sexual debut, prior screening for cervical cancer, history of abnormal Pap smears, sexually transmitted infections and genital warts. Furthermore, the referring gynecologist collected information on HPV vaccination status by checking the certificate of vaccination.

Participants underwent a pelvic examination with visualization of the uterine cervix. Pap smears were taken using spatula and endocervical brush. A second sample was then obtained with a Qiagen Cervical Sampler (Medscan, Uppsala, Sweden), and suspended in 1 ml of specimen transport medium (STM/Qiagen Inc., Hilden, Germany) for HPV DNA testing.

### HPV testing

All primary HPV testing was undertaken using the Hybrid Capture 2 (HC2) assay (HC2/Qiagen Inc., Hilden Germany). All samples were analyzed for the presence of at least 13 HR-HPV types (16, 18, 31, 33, 35, 39, 45, 51, 52, 56, 58, 59 and 68) following the manufacturer’s instructions. A positive HR-HPV result in this study refers to a subject positive for the HR-HPV probe mix. In this analysis, HR HPV subtypes were counted individually. In patients with multiple infections, HR HPV subtypes were reported separately. LR-HPV types (6, 11, 42, 43 and 44) are reported in a separate publication [12].

All samples that tested positive for HR-HPV with HC2 and 10% (every tenth) of all HC2-negative samples underwent HPV genotyping. HPV genotyping was performed as described previously using SPF-10-PCR, followed by Reverse Line Probe Assay LiPA Extra (SPF-10-PCR) [13]. Briefly, total DNA was isolated from the cervical samples with the use of a MagNAPure device (Roche, Indianapolis, IN) and analyzed with INNO-LiPA Extra HPV prototype assay (Innogenetics, Inc, Gent, Belgium) according to the manufacturer’s instructions. The INNO-LiPA Extra test identifies established HR-HPV types (16, 18, 31, 33, 35, 39, 45, 51, 52, 56, 58, 59 and 68 [1]) and five known or putative high-risk types (26, 53, 66, 73 and 82) [14,15]. Polymerase chain reaction (PCR) was performed according to good laboratory practice in a laboratory separate from other laboratory rooms. All PCR reactions were performed with 10 μl input DNA in a final volume of 50 μl using reagents provided by Innogenetics, 10 min 37°C, 9 min 94°C, and 40 cycles of 30 sec of denaturation at 94°C, followed by 45 sec of 52°C annealing temperature and 45 sec of extension at 72°C run on a MJ Thermocycler PCT 200. The PCR product was then denatured and a 10 μl aliquot hybridized to one strip at 49°C for 60 min, followed by multiple washing steps. The strips were analyzed on a flatbed scanner with the use of LiRAS prototype software (Innogenetics, Inc), which displays the patterns and relative intensity of positive bands as arbitrary grey-tone values between 0.1 and 1.0.

### Pap smears and colposcopy

A standardized, risk-adapted follow-up protocol was used for the management of participants according to the results of conventional Pap smear testing and HPV status, as described previously [16].

Patients were transferred for colposcopy if they had abnormal Pap smears conspicuous of high-grade lesions or had Pap smears classified as borderline/low-grade and tested positive for HR-HPV. Colposcopists classified the type of transformation zones according to the Barcelona nomenclature of the International Federation for Cervical Pathology and Colposcopy (IFCPC) [17]. Colposcopy findings were classified as minor changes (physiological changes and HPV infections with or without cervical intraepithelial neoplasia 1 [CIN1]) and major changes (CIN2+). For both categories separate measurements were made of the speed and intensity of acetowhite reactions and the morphology (condyloma like, flat, punctuation, mosaic). In cases of type 1 or type 2 transformation zone with visible squamous columnar junction, colposcopy was regarded as satisfactory. Any visible lesion underwent histological assessments with punch biopsies. No random punch biopsies were taken if colposcopy findings were normal. Histological assessment was mandatory for any lesion where there was a suspicion of high-grade neoplasia.

### Statistical analysis

This paper presents a one-time cross-sectional analysis of data for the 1983/84 cohort and baseline data for the 1988/89 cohort. As this is an observational study, no formal hypothesis was tested and the statistical analysis was descriptive for all evaluable variables. All statistical analyses were undertaken by an independent statistician who did not participate in the collection of data. The association between HR-HPV infection and exploratory variables was analyzed in univariate analysis with a level of significance defined as p < 0.05 (two-sided testing). Multivariate analyses include data on exploratory variables for all HPV subtypes. All statistical analyses were performed with the validated program Testimate Version 6.5 from IDV Gauting (validation of software, hardware and user according to FDA 21 CFR Part 11).

## Results

### Study population

Between 19 October 2009 and 31 December 2010, 659 (43.8%) of 1504 registered women born in 1983/84 and 599 (44.5%) of 1346 women born in 1988/89 were recruited. The characteristics and risk factors of interest for the two cohorts are shown in Table 1. In general, there were no notable differences between the two birth cohorts. The fact that patients in the 1988/89 cohort were younger than those 84 cohort,/84 cohort explains some of the numerical differences, such as in education, evident in Table 1.

**Table 1:** Patient characteristics, including risk factors

	**Cohort (n, unless stated otherwise)**^**a**^	
	**1983/84**	**1988/89**
Number recruited	659	599
Highest graduation		
None	21 (3%)	6 (1%)
Secondary school	354 (54%)	372 (62%)
Higher level	283 (43%)	221 (37%)
Country of birth		
Germany	474 (72%)	511 (85%)
Other	183 (28%)	88 (15%)
Stable relationship		
Yes	529 (80%)	423 (71%)
No	123 (19%)	169 (28%)
Current pregnancy		
Yes	27 (4%)	11 (2%)
No	627 (95%)	581 (97%)
Parity (mean ± SD)		
Pregnancies	0.7 ± 1.05	0.2 ± 0.56
Born children	0.5 ± 0.74	0.1 ± 0.36
Screening for cervical cancer		
Yes	548 (83%)	307 (51%)
Never	94 (14%)	274 (46%)
Contraception		
Hormonal^b^	679	590
Other^b^	517	457
None	23	21
Smoking history		
Current		
Yes	244 (37%)	221 (37%)
No	413 (63%)	377 (63%)
Former		
Yes	163 (25%)	74 (12%)
No	345 (52%)	392 (65%)
Number of sexual partners		
0	8 (1%)	30 (5%)
1	125 (19%)	126 (21%)
2–5	335 (51%)	336 (56%)
>5	162 (25%)	91 (15%)
Age at sexual debut (mean ± SD)	16.9 ± 2.45	16.1 ± 1.67
Pap smear test		
I	2 (<1%)	2 (<1%)
II	624 (95%)	579 (97%)
IIw	11 (2%)	12 (2%)
III	3 (<1%)	1 (<1%)
IIID	18 (3%)	5 (<1%)
Iva	1 (<1%)	–
History of sexually transmitted disease		
Yes	11 (2%)	4 (<1%)
No	648 (98%)	594 (99%)
History of genital warts		
Yes	26 (4%)	2 (<1%)
No	633 (96%)	163 (27%)
HPV vaccination		
No	616 (93%)	463 (77%)
Yes	42 (6%)	136 (23%)
Full 3 courses	40 (6%)	126 (21%)

^a^ Missing data are not included so n does not always add up to number recruited.

^b^ Multiple methods counted in the same individual.

### Prevalence of HR-HPV infection

**Figure 1:**
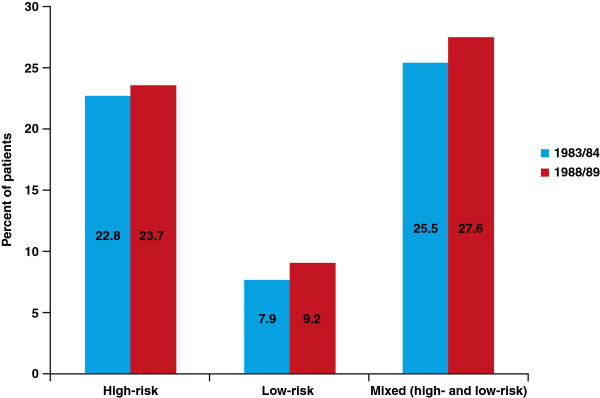
Positive HC2 test results for the 1983/84 and 1988/89 cohorts.

HC2 test results for HC2 HR-positive and LR-/HR-positive are shown in Figure 1. Overall, HR-HPV infection was identified by HC2 testing in 292/1258 women (23.2%; 95% confidence interval [CI] 20.9–25.6%). The prevalence of HR-HPV was 22.8% (95% CI 19.6–26.2%) 84 cohort,/84 cohort (150/659) and 23.7% (95% CI 20.4%–27.3%) in the 1988/99 cohort (142/599).

**Figure 2:**
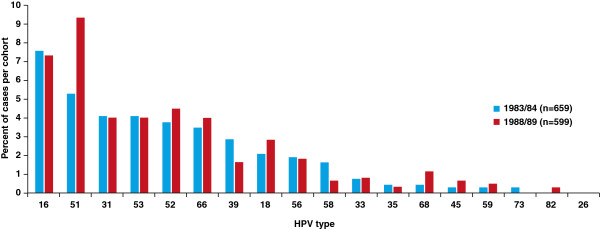
Type-specific prevalence of HR (16, 18, 31, 33, 35, 39, 45, 51, 52, 56, 58, 59, and 68) and putative HR (26, 53, 66, 73, and 82) HPV infection in 1983/84 and 1988/89 cohorts.

The type-specific HR-HPV prevalence is shown in Figure 2. In the 1983/84 cohort, the most frequent;HR-HPV types were 16 (7.59%) followed by types 51 (5.31%), 31 and 53 (both 4.1%), 52 (3.79%), 66 (3.49%), 39 (2.88%), and 18 (2.12%). In the 1988/89 cohort the most frequent HR-HPV types were 51 (9.35%), 16 (7.35%), 52 (4.51%), 31, 53 and 66 (all 4.01%), and 18 (2.84%).

### Cofactors associated with HR-HPV infection

**Figure 3:**
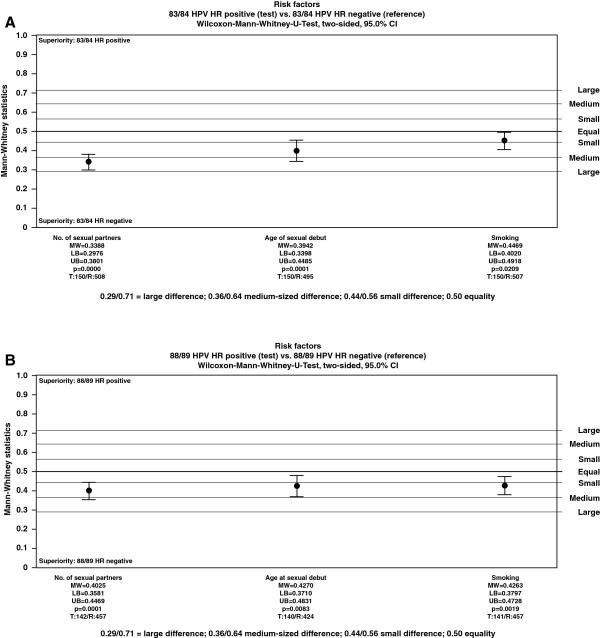
**Univariate analysis of risk factors for HR-HPV infection in (A) in 1983/84 cohort and (B) 1988/89 cohort.** In this analysis, the Mann Witney measure (0.0 to 1.0) reflects the probability that a randomly selected patient from the test group is better off than a randomly selected patient from the control group. Benchmark values are: 0.36 medium-sized inferiority, 0.44 small inferiority, 0.50 equality, 0.56 small superiority, 0.64 medium-sized (relevant) superiority, and 0.71 large superiority. For non-inferiority the benchmark is defined as MW-measure = 0.36 (medium-sized inferiority), for superiority the benchmark is defined as MW-measure = 0.50 (equality). The medium-sized superiority (inferiority) is – per definition – a medically relevant group-difference [18].

Univariate analysis showed that the number of sex partners (strong), early age at first intercourse (strong), and smoking (weak) were significant risk factors for HR-HPV infection in the 1983/84 cohort, whereas there were weaker associations in the 1988/89 cohort (Figure 3) [18]. Other parameters (contraception, history of STDs and age at first menstrual period) did not show any association with HR-HPV infection.

The three cofactors associated with HR-HPV infection were analyzed by specific HR-HPV type using the Mann Witney *U* test, two-sided for HPV-positive (test) versus HPV-negative (reference). In the 1983/84 cohort, the association between HPV16 infection and both the number of partners (>5 vs 1; p = 0.0000) and early age at first sex (p = 0.0065) was stronger than for HR-types overall. No association was evident in the 1988/89 cohort.

In multivariate analysis, only the number of sexual partners was a significant risk factor for HPV infection. The odds ratios (ORs) were 22.687 (95% CI 8.032–64.086) and 6.124 (95% CI 3.195–11.738) for more than five partners versus one partner in the 1983/84 and 1988/89 cohorts, respectively.

### Genotypes and atypical Pap smear test results

The association between atypical Pap smear test results and specific HR-HPV types is detailed in Table 2. HPV16 positive-women were significantly more likely to have abnormal Pap smears of any degree than were HPV16-negative women. In the 1983/84 cohort, the risk of > PapII (ASC-US [Atypical squamous cells of uncertain significance] or more) was 22.0% (95% CI 11.53–35.96%) in HPV16-positive women compared with 3.61% (95% CI 2.28–5.42%) in HPV16-negative women (p < 0.0001). In the 1988/89 cohort, the corresponding risks were 9.09% (95% CI 2.53 – 21.67%) in HPV16-positive women and 2.52% (95% CI 1.39–4.20%) in HPV16-negative women (p = 0.0482). Similarly, but in the 1983/84 cohort only, HPV16-positive women had a significantly increased risk for atypical Pap smears classified low-grade squamous intraepithelial lesion (LSIL) or more (p < 0.0001) and for biopsy proven CIN1+ (p < 0.0001).

**Table 2:** Atypical Pap smear test results and association with specific HR-HPV types

**HR-HPV type**^**a**^	**Prevalence of atypical Pap smear test results (%, 95% CIs) according to infection with specific HR-HPV types**							
	**1983/84 cohort (n = 659)**				**1988/89 cohort (n = 599)**			
	**HPV status**	**ASC-US or more**	**>LSIL**	**CIN1+**	**HPV status**	**ASC-US or more**	**>LSIL**	**CIN1+**
HPV16	Negative	3.61	2.30	1.31	Negative	2.52	0.90	0.90
	(n = 609)	(2.28-5.42)	(1.26-3.83)	(0.57-2.57)	(n = 555)	(1.39-4.20)	(0.29-2.09)	(0.29-2.09)
	Positive	22.00	16.00	14.00	Positive	9.09	2.27	4.55
	(n = 50)	(11.53-35.96)	(7.17-29.11)	(5.82-26.74)	(n = 44)	(2.53-21.67)	(0.06-12.02)	(0.56-15.47)
HPV18	Negative	4.50	2.95	2.02	Negative	2.75	1.03	1.03
	(n = 645)	(3.03-6.39)	(1.78-4.56)	(1.08-3.42)	(n = 582)	(1.58-4.43)	(0.38-2.23)	(0.38-2.23)
	Positive	28.57	21.43	14.29	Positive	11.76	0	5.88
	(n = 14)	(8.39-58.10)	(4.66-50.80)	(1.78-42.81)	(n = 17)	(1.46-36.44)	(0–19.51)	(0.15-28.69)
HPV31	Negative	5.22	3.48	2.37	Negative	2.78	1.04	1.04
	(n = 632)	(3.62-7.26)	(2.19-5.22)	(1.33-3.88)	(n = 575)	(1.60-4.48)	(0.38-2.26)	(0.38-2.26)
	Positive	0	0	0	Positive	8.33	0	4.17
	(n = 27)	(0–1.28)	(0–1.28)	(0–12.77)	(n = 24)	(1.03-27.00)	(0–14.25)	(0.11-21.12)
HPV33	Negative	5.05	3.36	2.29	Negative	3.03	1.01	1.18
	n = 654)	(3.50-7.01)	(2.11-5.05)	(1.29-3.75)	(n = 594)	(1.81-4.75)	(0.37-2.19)	(0.48-2.41)
	Positive	0	0	0	Positive	0	0	0
	(n = 5)	(0–52.18)	(0–52.18)	(0–52.18)	(n = 5)	(0–52.18)	(0–52.18)	(0–52.18)
HPV35	Negative	4.88	3.35	2.13	Negative	3.02	1.01	1.17
	(n = 656)	(3.36-6.82)	(2.11-5.03)	(1.17-3.55)	(n = 597)	(1.80-4.72)	(0.37-2.17)	(0.47-2.40)
	Positive	33.33	0	33.33	Positive	0	0	0
	(n = 3)	(0.84-90.57)	(0–70.76)	(0.84-90.57)	(n = 2)	(0–84.19)	(0–84.19)	(0–84.19)
HPV39	Negative	4.69	2.97	2.19	Negative	3.06	1.02	1.19
	(n = 640)	(3.18-6.62)	(1.80-4.60)	(1.20-3.64)	(n = 589)	(1.82-4.79)	(0.37-2.20)	(0.48-2.43)
	Positive	15.79	15.79	5.26	Positive	0	0	0
	(n = 19)	(3.38-39.58)	(3.38-39.58)	(0.13-26.03)	(n = 10)	(0–30.85)	(0–30.85)	(0–30.85)
HPV51	Negative	4.49	2.72	1.76	Negative	2.58	0.55	1.10
	(n = 624)	(3.00-6.42)	(1.59-4.33)	(0.88-3.13)	(n = 543)	(1.42-4.29)	(0.11-1.61)	(0.41-2.39)
	Positive	14.29	14.29	11.43	Positive	7.14	5.36	1.79
	(n = 35)	(4.81-30.26)	(4.81-30.26)	(3.20-26.74)	(n = 56)	(1.98-17.29)	(1.12-14.87)	(0..05-9.55)
HPV52	Negative	4.89	3.15	2.21	Negative	2.80	0.70	1.05
	(n = 634)	(3.35-6.87)	(1.94-4.83)	(1.21-3.68)	(n = 572)	(1.61-4.50)	(0.19-1.78)	(0.39-2.27)
	Positive	8.00	8.00	4.00	Positive	7.41	7.41	3.70
	(n = 25)	(0.98-26.03)	(0.98-26.03)	(0.10-20.35)	(n = 27)	(0.91-24.29)	(0.91-24.29)	(0.09-18.97)
HPV53	Negative	4.43	2.85	2.06	Negative	2.78	0.87	1.22
	(n = 632)	(2.96-6.34)	(1.70-4.46)	(1.10-3.49)	(n = 575)	(1.60-4.48)	(0.28-2.02)	(0.49-2.49)
	Positive	18.52	14.81	7.41	Positive	8.33	4.17	0
	(n = 27)	(6.30-38.08)	(4.19-33.73)	(0.91-24.29)	(n = 24)	(1.03-27.00)	(0.11-21.12)	(0–14.25)
HPV66	Negative	4.25	2.67	1.89	Negative	2.78	0.87	1.04
	(n = 636)	(2.82-6.12)	(1.56-4.25)	(0.98-3.27)	(n = 575)	(1.6-4.48)	(0.28-2.02)	(0.38-2.26)
	Positive	26.09	21.74	13.04	Positive	8.33	4.17	4.17
	(n = 23)	(10.23-48.41)	(7.46-43.70)	(2.78-33.59)	(n = 24)	(1.03-27.00)	(0.11-21.12)	(0.11-21.12)

^a^HPV subtypes were counted individually. In patients with multiple HR HPV infections, the data are reported for each subtype separately, e.g. a mixed HPV16/52 infection is included in the rows for both HPV16 and for HPV52.

In HPV18-positive women in the 1983/84 cohort, the corresponding risks were significantly increased for atypical Pap smears classified borderline or more (p < 0.0034), LSIL or more (p < 0.0088) and CIN1+ (p < 0.04). No significant differences were observed in the 1988/89 cohort.

Type-specific univariate analyses of data from the 1983/84 cohort showed significant interactions between other specific HPV types and atypical Pap smear test results. There was a weak association between HPV39 and Pap LSIL or more (p < 0.0239). HPV51 showed a weak association with Pap ASC-US or more (p < 0.0318), but a stronger association with Pap smears LSIL or more (p < 0.0047) and histology of CIN1+ (p < 0.0066). HPV53 showed a significant association with Pap ASC-US or more (p < 0.0093) and LSIL or more (p < 0.0107), but not with CIN1+. HPV66 showed significant associations with Pap ASC-US + (p < 0.0005), LSIL (p < 0.0005) and CIN1+ (p < 0.0134). HPV types 31, 33, 35, 45 and 52 showed no associations at all.

For the 1988/89 cohort, type-specific univariate analysis found no association between HPV types 31, 33, 35, 39, 45, 51, 53 and 66 and atypical smear tests; however, there was a significant association between HPV52 (n = 27) and Pap smears LSIL or more (p < 0.0284)

### CIN2 and CIN3 and associated HPV types

Overall eight women were diagnosed with CIN3, six in the 1983/84 cohort and two in the 1988/89 cohort. All women with CIN3 tested positive for HC2-HR. Genotyping showed that all six CIN3 cases in the 1983/84 cohort tested positive for HPV16 (as single infections in two cases and as co-infections with HPV51 in two cases, HPV52 in one case and HPV35 in one case). For HPV16-infected women in the 1983/84 cohort the corresponding risk for CIN3 was 6/50 (12%). In the 1988/89 cohort, genotyping showed one case of co-infection with HPV16 and HPV52, but failed in the other case.

Overall 11 women were diagnosed with CIN2, six in the 1983/84 cohort and five in the 1988/89 cohort. All tested positive for HC2-HR. Genotyping showed that three cases of CIN2 in the 1983/84 cohort were associated with HPV16 (one HPV16, 39 and 66, one HPV16, 52 and 66 and one HPV16 and 66) while the remaining cases were associated with HPV18 (n = 1), HPV18 and 31 (n = 1) and HPV53 (n = 1). In the 1988/89 cohort, genotyping showed four cases of CIN2 were associated with HPV16 (two HPV16 only, two HPV16 and 66) and one case was associated with HPV51.

### History of cone biopsies/excisional treatment

Five women in the 1983/84 cohort had a history of conization because of CIN3, whereas there were no cases with a history of conization in the 1988/89 cohort.

### Colposcopy morphology and association with HPV types

In total, 46 women were referred to colposcopy, all of whom had transformation zones either type 1 (n = 28) or type 2 (n = 18). Colposcopy was considered to be satisfactory in all of these cases. Colposcopy findings were classified as major changes in 11 cases, minor changes in 25 cases and as normal in 10 cases.

All eight CIN3 lesions showed major changes with fast acetic acid reaction, dense acetowhite to opaque flat epithelium (n = 4), coarse mosaic (n = 1), coarse punctuation (n = 2) or coral-like with internal borders (n = 1). All CIN3 lesions were associated with HPV16.

Only three CIN2 lesions showed major changes with coarse punctuation (n = 2) or flat dense acetowhite (n = 1), and all were associated with HPV16 or HPV18, while the remaining eight CIN2 lesions showed minor changes, mainly regular punctuation and flat acetowhite epithelium. Among cases with normal histology or CIN1 and minor changes (n = 27), no association was demonstrated between specific HPV types and defined morphological changes on colposcopy.

### HPV vaccination

Analysis of HPV vaccination was not useful in the 1983/84 cohort because of the low number of vaccinated participants (42/659). However, 126 of 599 women in the 1988/89 cohort had received a full course of three doses of HPV vaccines, which was more than expected at the start of the study. Only two of 126 were diagnosed with HPV16 infection (1.59%) compared with 42 of 476 non-vaccinated women (8.82%); the difference was statistically significant (p = 0.003). There was no CIN associated with HPV 16 or 18 in the vaccinated group.

## Discussion

WOLVES has provided the first real-life data in Germany on the changing dynamics of HR-HPV and associated diseases in women aged 20–30 years who have not been routinely vaccinated. Essentially, the study represents the final opportunity to monitor a population of young women at full risk from HPV and provides benchmark longitudinal data that will allow the impact of HPV vaccination to be measured. The overall prevalence of HR-HPV in this study was 23%. HPV16 was the most common type in 1983/84 cohort and the second most common type after HPV51 in the 1988/89 cohort. The risk for HR-HPV infection was increased by the number of sexual partners, age at sexual debut and smoking (weak association). Multivariate analysis demonstrated that only the number of sexual partners was a significant risk factor for HPV infection. This finding supports the concept that the number of sexual partners is the single most important risk factor for HPV infection, although other factors may be more important for HPV persistency [19]. Hence, the overall risk of cancer may be more dependent on cofactors that compromise the interaction between host and virus [20].

Other national studies in Europe, using heterogeneous sources and methodologies, have provided HPV prevalence data in women aged <30 years prior to widespread HPV vaccination. In a German cross-sectional study [10], 377/1692 (22.3%) women aged 10–30 years had positive HC2-HR results (including mixed LR and HR), of whom 239 had HR-HPV only (14.1%). In the age group 20–22 years rates were 28.3% for LR/HR and 15.3% for HR only; 95.8% of women did not show signs of any cervical lesion on cytology. Adjusted analysis identified the number of sexual partners (OR: 1.105 [95% CI 1.069–1.142]), smoking (OR: 1.508 [1.155-1.968]), and vaccination against HPV (OR: 0.589 [0.398-0.872]) rather than increasing age as risk factors associated with HPV infection. A UK study used samples from the National Chlamydia Screening Program (NCSP) and Prevention of Pelvic Infection (POPI) trial [21]. Of 3829 samples, 3554 (2369 + 1185) were from women aged 16–24 years. The prevalence of HR-HPV infection was 34.6% (17.6% HPV16 and 18) in NCSP and 18.2% (7.2% HPV16 and 18) in POPI. The risk of HR-HPV infection was increased in women who reported at least two sexual partners. The CLEOPATRE study included women aged 18–64 years in Spain and Portugal [22,23]. The highest prevalence of HR-HPV was observed in women aged 18–24 years (27.0–28.8%) [23]. The lifetime number of sexual partners was a strong predictor of HPV infection (OR 5.44 for 5–10 partners versus one partner; p < 0.001).

In other European studies of non-vaccinated women aged 18 to <30 years, the prevalence of HR-HPV varied considerably from <10% to >50% [24-32]. It is important to note that observed prevalence rates are highly dependent on the methods used for HPV testing. Nevertheless, overall, these data show that HR-HPV infection is relatively common in young women who have not received HPV vaccination and that the number of sexual partners is consistently a strong risk factor for infection. From a clinical perspective, there is still a significant population of young women infected with HR-HPV who will have a higher rate of cervical screening abnormalities and, ultimately, an increased risk of developing cancer.

With its well-defined target population, the ongoing WOLVES study has a number of advantages compared with one-time, cross-sectional studies in mixed screening populations. The 1988/89 cohort will be followed by annual examination for 5 years. In 2014/15, women born in 1993/94 will be invited for a one-time examination. WOLVES will finally comprise three different age cohorts (women born in 1983/84, 1988/89 and 1993/94) allowing a comparison of changes over time. At the end of the trial in 2014/15, the 1993/94 cohort will be as old as the 1988/89 cohort was at the beginning of the trial in 2009, while the 1988/89 cohort will be as old as the 1983/84 cohort was in 2009.

A particular strength of the WOLVES study design is careful monitoring of defined patient pathways in participants with abnormal screening results or clinical findings. As a result, compliance was high and almost 90% of referred participants underwent a colposcopy examination. A strong association (Mann Whitney 0.3388) was found between HPV16 infection and ASC-US (>PapII), LISL, and CIN + in 1983/84 cohort, but only for ASC-US (>PapII) in 1988/89 cohort. This age-related pattern is probably explained by a high persistency rate and increased risk of oncogenic transformation over time, consistent with observations reported by Kjaer and colleagues showing that the increased risk of CIN3 or cancer associated with HR-HPV infection, especially HPV16, is a continuous trend over several decades [33]. Furthermore, data from the HERACLES and SCALE studies have confirmed the predominant importance of HPV16 infection in both high-grade CIN and invasive cervical cancer [34]. Along with findings from a recent analysis that demonstrated a higher prevalence of HPV16 associated with younger age in women with CIN3 [35], the data from the WOLVES study fit perfectly with the proposed theory that HPV16 infection is the predominant cause of CIN3 in young women. In the WOLVES study, other HR-HPV types (39, 51, 53 and 66) showed weak associations with abnormal Pap findings and are unlikely to be clinically relevant. It remains important to assess risk accurately so that clinically irrelevant abnormal Pap findings do not lead to an increased risk of over-diagnosis and over-treatment in young women [16].

An association between HPV52 and LSIL or more was observed in the 1988/89 cohort, but not in 1983/84 cohort. LSIL can result from harmless transient HPV infections and the difference between cohorts is most likely to be a chance finding. An imbalance in conization history between the 1983/84 and 1988/89 cohorts can be explained by the conservative policy adopted in the colposcopy clinic in Wolfsburg, which has been the referral center for most cases of abnormal smears in the city for more than 8 years. The corresponding figures in other German regions may be significantly higher.

Correlative data for HPV infection and associated pathological abnormalities will help to further refine the clinical management of young women at risk for malignancies associated with HR-HPV infection, especially HPV16. The data from the WOLVES study support recommendations for a later start of cervical screening, at the age of 25 years or later, no routine HPV testing in women younger than 25 years and conservative management of individuals presenting with abnormal Pap smears. In addition, preliminary data from the WOLVES study showed that HPV vaccination significantly reduces the rate of HPV16 infection, corroborating recent data from a national study in Australia [36].

A limitation of this first analysis of the WOLVES study is that it provides only prevalence data and as yet does not show the dynamics of HPV infection and changes in pathology findings over time. Nevertheless, the WOLVES data are in accordance with published data from similar European trials of HPV types commonly associated with atypical Pap smears/cervical neoplasia [28,29,37,38] and support the concept that HR-HPV infections leading to the development of cancer are acquired early in young women who have not been vaccinated. A planned analysis in 2014/15 will provide data on the dynamics of HPV infection and additional correlative clinical findings in a population at full risk from HR-HPV-related malignant disease.

## Conclusions

Only a high number of sexual partners (>5) was an independent risk factor for HPV infection. The effect of cofactors for the risk of CIN3 and cancer, such as smoking and use of contraceptives, may not yet be apparent because these factors probably trigger HPV persistency rather than acquisition. There was a high prevalence of HR-HPV infection in women aged 20 and 25 years in 2009, which was associated with an increased risk of abnormal Pap smears and biopsy proven CIN2+. Women with HPV16 infection have a high risk of clinically relevant lesions that seems to increase over time (as the comparison of the two age cohorts shows) and there was a significant impact of HPV vaccination on HPV16 infections, although the majority of these women must have been sexually active already at the time of vaccination and, therefore, the efficiency must be considered to be suboptimal.

## Abbreviations

ASC-US: Atypical squamous cells of uncertain significance; CIN: Cervical intraepithelial neoplasia; HC2: Hybrid capture 2; HR-HPV: High-risk human papilloma virus; LR-HPV: Low-risk human papilloma virus; LSIL: Low-grade squamous intraepithelial lesion; Pap: Papanicolaou test; PCR: Polymerase chain reaction; WOLVES: Wolfsburg HPV epidemiological study

## Competing interests

WOLVES is financed mainly by an unrestricted grant from Sanofi Pasteur MSD and supported by free samplers distributed by Qiagen and Hologic. KUP received speaker´s honorarium from Beckton Dickinson, Qiagen and Roche Diagnostics. RS-R is employee of Sanofi Pasteur MSD. TI has received institutional grants from GSK, Gen-Probe, Sanofi-Pasteur MSD and Hologic. The remaining authors declare they have no competing interests.

## Authors’ contributions

KUP was responsible for the overall study design, data analysis, interpretation, writing of the manuscript and data collection. AL was responsible for data collection and interpretation. AJ was responsible for statistical analyses and interpretation. AI was responsible for HC2-testing, HPV genotyping and sequencing, data collection and interpretation. SS was responsible for trial coordination, quality control and data collection. AR-L was responsible for histology. RSR was responsible for trial coordination and interpretation. EG was responsible for recruitment, data collection and interpretation. TI was responsible for the study design, HPV testing, data collection and interpretation. All authors read and approved the final manuscript.

## Pre-publication history

The pre-publication history for this paper can be accessed here:

http://www.biomedcentral.com/1471-2334/13/135/prepub

## References

[B1] Schiffman M Clifford G Buonaguro FM Classification of weakly carcinogenic human papillomavirus types: addressing the limits of epidemiology at the borderline Infect Agent Cancer 2009 4 8 10.1186/1750-9378-4-8 19486508 PMC2694995

[B2] Bosch FX de Sanjosé S Human papillomavirus and cervical cancer – burden and assessment of causality J Natl Cancer Inst Monogr 2003 31 3 13 12807939 10.1093/oxfordjournals.jncimonographs.a003479

[B3] Pathirana D Hillemanns P Petry KU Becker N Brockmeyer NH Erdmann R Gissmann L Grundhewer H Ikenberg H Kaufmann AM Klusmann J Kopp I Pfister H Rzany B Schneede P Schneider A Smola S Winter-Koch N Wutzler P Gross G Short version of the German evidence-based guidelines for prophylactic vaccination against HPV-associated neoplasia Vaccine 2009 27 4551 4559 10.1016/j.vaccine.2009.03.086 19524337

[B4] Markowitz LE Dunne EF Saraiya M Lawson HW Chesson H Unger ER Centers for Disease Control and Prevention (CDC) Quadrivalent human papillomavirus vaccine: recommendations of the advisory committee on immunization practices (ACIP) MMWR Recomm Rep 2007 56 RR-2 1 24 17380109

[B5] European Centre for Disease Prevention and Control Introduction of HPV vaccines in EU countries – an update 2012 Stockholm: ECDC

[B6] Hariri S Markowitz L Monitoring HPV vaccine impact: early results and ongoing challenges J Infect Dis 2012 206 1633 1635 10.1093/infdis/jis593 23087429

[B7] Klug SJ Hukelmann M Hollwitz B Düzenli N Schopp B Petry KU Iftner T Prevalence of human papillomavirus types in women screened by cytology in Germany J Med Virol 2007 79 616 625 10.1002/jmv.20863 17385693

[B8] Luyten A Theiler KG Pietralla M Braun BE Reinecke-Lüthge A Petry KU Primary HPV-screening project in Wolfsburg, Germany. Experience over 18 months Geburtsh Frauenheilk 2008 68 273 10.1016/S1386-6532(09)70294-X 20129072

[B9] Petry KU Menton S Menton M van Loenen-Frosch F de Carvalho Gomes H Holz B Schopp B Garbrecht-Buettner S Davies P Boehmer G van den Akker E Iftner T Inclusion of HPV testing in routine cervical cancer screening for women above 29 years in Germany: results for 8466 patients Br J Cancer 2003 88 1570 1577 10.1038/sj.bjc.6600918 12771924 PMC2377109

[B10] Iftner T Eberle S Iftner A Holz B Banik N Quint W Straube AN Prevalence of low-risk and high-risk types of human papillomavirus and other risk factors for HPV infection in Germany within different age groups in women up to 30 years of age: an epidemiological observational study J Med Virol 2010 82 1928 1939 10.1002/jmv.21910 20872721

[B11] Petry KU Breugelmans JG Benard S Lamure E Littlewood KJ Hillemanns P Cost of screening and treatment of cervical dyskaryosis in Germany Eur J Gynaecol Oncol 2008 29 345 349 18714567

[B12] Petry KU Luyten A Justus A Iftner A Strehlke S Schulze-Rath R Iftner T **Prevalence of low-risk HPV types and genital warts in women born 1988/89 or 1983/84 – results of WOLVES, a population-based epidemiological study in Wolfsburg, Germany** BMC Infect Dis 2012 12 367 10.1186/1471-2334-12-367 23259726 PMC3536688

[B13] Klug SJ Molijn A Schopp B Holz B Iftner A Quint W Snijders PJF Petry KU Krüger Kjaer S Munk C Iftner T Comparison of the performance of different HPV genotyping methods for detecting genital HPV types J Med Virol 2008 80 1264 1274 10.1002/jmv.21191 18461626

[B14] Cogliano V Baan R Straif K Grosse Y Secretan B El GF Carcinogenicity of human papillomaviruses Lancet Oncol 2005 6 204 10.1016/S1470-2045(05)70086-3 15830458

[B15] Muñoz N Bosch FX de Sanjosé S Herrero R Castellsagué X Shah KV Snijders PJ Meijer CJ International Agency for Research on Cancer Multicenter Cervical Cancer Study Group Epidemiologic classification of human papillomavirus types associated with cervical cancer N Engl J Med 2003 348 518 527 10.1056/NEJMoa021641 12571259

[B16] Luyten A Scherbring S Reinecke-Lüthge A Braun BE Pietralla M Theiler K Petry KU Risk-adapted primary HPV cervical cancer screening project in Wolfsburg, Germany-experience over 3 years J Clin Virol 2009 46 Suppl 3 S5 10 20129072 10.1016/S1386-6532(09)70294-X

[B17] Walker P Dexeus S De Palo G Barrasso R Campion M Girardi F Jakob C Roy M International terminology of colposcopy: an updated report from the international federation for cervical pathology and colposcopy Obstet Gynecol 2003 101 175 177 10.1016/S0029-7844(02)02581-4 12517664

[B18] Colditz GA Miller JN Mosteller F Measuring gain in the evaluation of medical technology. The probability of a better outcome Int J Technol Assess Health Care 1988 4 637 642 10.1017/S0266462300007728 10291102

[B19] Oakeshott P Aghaizu A Reid F Howell-Jones R Hay PE Sadiq ST Lacey CJ Beddows S Soldan K Frequency and risk factors for prevalent, incident, and persistent genital carcinogenic human papillomavirus infection in sexually active women: community based cohort study BMJ 2012 344 e4168 10.1136/bmj.e4168 22730542 PMC3382227

[B20] Frazer IH Interaction of human papillomaviruses with the host immune system: a well evolved relationship Virology 2009 384 410 414 10.1016/j.virol.2008.10.004 18986661

[B21] Howell-Jones R de Silva N Akpan M Oakeshott P Carder C Coupland L Sillis M Mallinson H Ellis V Frodsham D Robinson TI Gill ON Beddows S Soldan K Prevalence of human papillomavirus (HPV) infections in sexually active adolescents and young women in England, prior to widespread HPV immunisation Vaccine 2012 30 3867 3875 10.1016/j.vaccine.2012.04.006 22516212

[B22] Pista A de Oliveira CF Cunha MJ Paixao MT Real O CLEOPATRE Portugal Study Group Risk factors for human papillomavirus infection among women in Portugal: the CLEOPATRE Portugal study Int J Gynaecol Obstet 2012 118 112 116 10.1016/j.ijgo.2012.03.028 22608026

[B23] Pista A de Oliveira CF Cunha MJ Paixao MT Real O CLEOPATRE Portugal Study Group Prevalence of human papillomavirus infection in women in Portugal: the CLEOPATRE Portugal study Int J Gynecol Cancer 2011 21 1150 1158 10.1097/IGC.0b013e31821dd3b2 21792018

[B24] Giorgi Rossi P Bisanzi S Paganini I Di Iasi A Angeloni C Scalisi A Macis R Pini MT Chini F Carozzi FM HPV Prevalence Italian Working Group Prevalence of HPV high and low risk types in cervical samples from the Italian general population: a population based study BMC Infect Dis 2010 10 214 20646310 10.1186/1471-2334-10-214 PMC2916912

[B25] Piana A Sotgiu G Castiglia P Pischedda S Cocuzza C Capobianco G Marras V Dessole S Muresu E Prevalence and type distribution of human papillomavirus infection in women from north Sardinia Italy BMC Publ Health 2011 11 785 10.1186/1471-2458-11-785 PMC3208589 21989375

[B26] Johnson AM Mercer CH Beddows S de Silva N Desai S Howell-Jones R Carder C Sonnenberg P Fenton KA Lowndes C Soldan K Epidemiology of, and behavioural risk factors for, sexually transmitted human papillomavirus infection in men and women in Britain Sex Transm Infect 2012 88 212 217 10.1136/sextrans-2011-050306 22261135 PMC3308471

[B27] Arbyn M Benoy I Simoens C Bogers J Beutels P Depuydt C Prevaccination distribution of human papillomavirus types in women attending at cervical cancer screening in Belgium Cancer Epidemiol Biomarkers Prev 2009 18 321 330 10.1158/1055-9965.EPI-08-0510 19124515

[B28] Sargent A Bailey A Almonte M Turner A Thomson C Peto J Desai M Mather J Moss S Roberts C Kitchener HC ARTISTIC Study Group Prevalence of type-specific HPV infection by age and grade of cervical cytology: data from the ARTISTIC trial Br J Cancer 2008 98 1704 1709 10.1038/sj.bjc.6604324 18392052 PMC2391119

[B29] Cuschieri KS Cubie HA Whitley MW Seagar AL Arends MJ Moore C Gilkisson G McGoogan E Multiple high risk HPV infections are common in cervical neoplasia and young women in a cervical screening population J Clin Pathol 2004 57 68 72 10.1136/jcp.57.1.68 14693839 PMC1770158

[B30] Lenselink CH Melchers WJ Quint WG Hoebers AM Hendriks JC Massuger LF Bekkers RL Sexual behaviour and HPV infections in 18 to 29 year old women in the pre-vaccine era in the Netherlands PLoS One 2008 3 e3743 10.1371/journal.pone.0003743 19011683 PMC2581437

[B31] Schmeink CE Melchers WJ Siebers AG Quint WG Massuger LF Bekkers RL Human papillomavirus persistence in young unscreened women, a prospective cohort study PLoS One 2011 6 e27937 10.1371/journal.pone.0027937 22132173 PMC3223200

[B32] De Vuyst H Clifford G Li N Franceschi S HPV infection in Europe Eur J Cancer 2009 45 2632 2639 10.1016/j.ejca.2009.07.019 19709878

[B33] Kjær SK Frederiksen K Munk C Iftner T Long-term absolute risk of cervical intraepithelial neoplasia grade 3 or worse following human papillomavirus infection: role of persistence J Natl Cancer Inst 2010 102 1478 1488 10.1093/jnci/djq356 20841605 PMC2950170

[B34] Tjalma WA Fiander A Reich O Powell N Nowakowski AM Kirschner B Koiss R O'Leary J Joura EA Rosenlund M Colau B Schledermann D Kukk K Damaskou V Repanti M Vladareanu R Kolomiets L Savicheva A Shipitsyna E Ordi J Molijn A Quint W Raillard A Rosillon D De Souza SC Jenkins D Holl K for the HERACLES/SCALE Study Group Differences in human papillomavirus type distribution in high-grade cervical intraepithelial neoplasia and invasive cervical cancer in Europe Int J Cancer 2013 132 4 854 67 Epub 2012 Jul 24 10.1002/ijc.27713 22752992

[B35] Brotherton JM Tabrizi SN Garland SM Does HPV type 16 or 18 prevalence in cervical intraepithelial neoplasia grade 3 lesions vary by age? an important issue for postvaccination surveillance Future Microbiol 2012 7 193 199 10.2217/fmb.11.161 22324989

[B36] Tabrizi SN Brotherton JM Kaldor JM Skinner SR Cummins E Liu B Bateson D McNamee K Garefalakis M Garland SM Fall in human papillomavirus prevalence following a national vaccination program J Infect Dis 2012 206 1645 1651 10.1093/infdis/jis590 23087430

[B37] Brismar-Wendel S Froberg M Hjerpe A Andersson S Johansson B Age-specific prevalence of HPV genotypes in cervical cytology samples with equivocal or low-grade lesions Br J Cancer 2009 101 511 517 10.1038/sj.bjc.6605165 19623178 PMC2720239

[B38] Howell-Jones R Bailey A Beddows S Sargent A de Silva N Wilson G Anton J Nichols T Soldan K Kitchener H Study Group Collaborators Multi-site study of HPV type-specific prevalence in women with cervical cancer, intraepithelial neoplasia and normal cytology, in England Br J Cancer 2010 103 209 216 10.1038/sj.bjc.6605747 20628396 PMC2906740

